# Coactivation of GSK3β and IGF-1 Attenuates Amyotrophic Lateral Sclerosis Nerve Fiber Cytopathies in SOD1 Mutant Patient-Derived Motor Neurons

**DOI:** 10.3390/cells10102773

**Published:** 2021-10-16

**Authors:** Hsiao-Chien Ting, Hui-I Yang, Horng-Jyh Harn, Ing-Ming Chiu, Hong-Lin Su, Xiang Li, Mei-Fang Chen, Tsung-Jung Ho, Ching-Ann Liu, Yung-Jen Tsai, Tzyy-Wen Chiou, Shinn-Zong Lin, Chia-Yu Chang

**Affiliations:** 1 Bioinnovation Center, Buddhist Tzu Chi Medical Foundation, Hualien 97002, Taiwan; sharkzoe@yahoo.com.tw (H.-C.T.); s8706083@yahoo.com.tw (H.-I.Y.); arthewduke@gmail.com (H.-J.H.); sagianne@gmail.com (C.-A.L.); cljbalaa@gmail.com (Y.-J.T.); 2 Institute of Medical Sciences, Tzu Chi University, Hualien 97004, Taiwan; jeron888@gmail.com; 3 Department of Pathology, Hualien Tzu Chi Hospital and Tzu Chi University, Hualien 97002, Taiwan; 4 Institute of Cellular and System Medicine, National Health Research Institutes, Miaoli 35053, Taiwan; ingming@nhri.edu.tw; 5 Department of Life Sciences, National Chung Hsing University, Taichung 40227, Taiwan; suhonglin@gmail.com; 6 Waisman Center, University of Wisconsin, Madison, WI 53705, USA; michael2025li@163.com; 7 Department of Medical Research, Hualien Tzu Chi Hospital, Hualien 97002, Taiwan; mfchen@mail.tcu.edu.tw; 8 Department of Chinese Medicine, Hualien Tzu Chi Hospital, Hualien 97002, Taiwan; 9 Integration Center of Traditional Chinese and Modern Medicine, Hualien Tzu Chi Hospital, Hualien 97002, Taiwan; 10 School of Post-Baccalaureate Chinese Medicine, Tzu Chi University, Hualien 97004, Taiwan; 11 Neuroscience Center, Hualien Tzu Chi Hospital, Hualien 97002, Taiwan; 12 Department of Life Science, National Dong Hwa University, Hualien 97441, Taiwan; twchiou@gms.ndhu.edu.tw; 13 Department of Neurosurgery, Hualien Tzu Chi Hospital, Hualien 97002, Taiwan

**Keywords:** amyotrophic lateral sclerosis (ALS), induced pluripotent stem cell (iPSC), motor neuron (MN), *SOD1*, drug screening, gastrodin, GSK3β, IGF-1

## Abstract

Amyotrophic lateral sclerosis (ALS) is a progressive nervous system disease that causes motor neuron (MN) degeneration and results in patient death within a few years. To recapitulate the cytopathies of ALS patients’ MNs, *SOD1*^G85R^ mutant and corrected *SOD1*^G85G^ isogenic-induced pluripotent stem cell (iPSC) lines were established. Two *SOD1* mutant ALS (*SOD1^G85R^* and *SOD1*^D90A^), two *SOD1* mutant corrected (*SOD1*^G85G^ and *SOD1*^D90D^), and one sporadic ALS iPSC lines were directed toward MNs. After receiving ~90% purity for MNs, we first demonstrated that *SOD1*^G85R^ mutant ALS MNs recapitulated ALS-specific nerve fiber aggregates, similar to *SOD1*^D90A^ ALS MNs in a previous study. Moreover, we found that both *SOD1* mutant MNs showed ALS-specific neurite degenerations and neurotransmitter-induced calcium hyperresponsiveness. In a small compound test using these MNs, we demonstrated that gastrodin, a major ingredient of *Gastrodia elata*, showed therapeutic effects that decreased nerve fiber cytopathies and reverse neurotransmitter-induced hyperresponsiveness. The therapeutic effects of gastrodin applied not only to *SOD1* ALS MNs but also to sporadic ALS MNs and *SOD1*^G93A^ ALS mice. Moreover, we found that coactivation of the GSK3β and IGF-1 pathways was a mechanism involved in the therapeutic effects of gastrodin. Thus, the coordination of compounds that activate these two mechanisms could reduce nerve fiber cytopathies in *SOD1* ALS MNs. Interestingly, the therapeutic role of GSK3β activation on *SOD1* ALS MNs in the present study was in contrast to the role previously reported in research using cell line- or transgenic animal-based models. In conclusion, we identified in vitro ALS-specific nerve fiber and neurofunctional markers in MNs, which will be useful for drug screening, and we used an iPSC-based model to reveal novel therapeutic mechanisms (including GSK3β and IGF-1 activation) that may serve as potential targets for ALS therapy.

## 1. Introduction

Amyotrophic lateral sclerosis (ALS) is one of the most serious motor neuron (MN) degenerative diseases: It has an incidence of five to six cases per million people and an effective treatment is not currently available. ALS patients suffer from motor function degeneration from the body terminals; within 3–5 years, patients lose most of their motor function and finally die from respiratory failure [1]. Current FDA-approved ALS treatment drugs, including riluzole, edaravone, and Nuedexta, which show limited therapeutic effects on ALS patients [2,3,4].

Most ALS cases are sporadic with unknown causes, whereas 10–20% are from pathogenic genetic mutations. Known mutations of genes such as *SOD1* [5,6], *ALS2* [7], *TARDBP* [8,9], *C9orf72* [10], *FUS* [11,12], *OPTN* [13], and *VAPB* [14] induce familial or early-onset ALS. About 20% of familial ALS cases are caused by *SOD1* mutations [5,6].

The clinical pathology of ALS includes mutated pathogenic protein accumulation, nerve fiber degeneration, and MN functional loss [15,16]. Other cytopathies, including endoplasmic reticulum stress, mitochondrial dysfunction, and high-level reactive oxygen species, have been identified in various ALS in vitro and animal models [17,18,19]. These MN cytopathies can lead to the disruption of neuron–neuron and neuromuscular junctions.

Although transgenic cell lines and animals have been used in ALS research for decades, huge gaps still exist between current laboratory models and human patients. Most models have been generated from immortalized cell lines or animals with high-level expression of human ALS pathogenic transgenes [20,21]. Thus, these models may have ALS like late-stage outcomes but dissimilar disease mechanisms compare with those of real patients. To recapitulate the cytopathies of patients, patient cell-derived induced pluripotent stem cells (iPSCs) are now applied in ALS in vitro modeling. Neurite length decrease, protein aggregates, oxidative stress, mitochondrial dysfunction, and cell apoptosis have all been observed in non-*SOD1* ALS MNs [22,23,24,25,26,27,28,29,30,31,32,33]. *SOD1* mutation MNs express SOD1 aggregates and neurofilament dysregulation phenotypes [27]. Until now, two potential compounds, Src/c-Abl inhibitor and ropinirole, have been screened from ALS iPSC-based models and are under clinical development [26,32]. However, ropinirole exhibits no significant effects on *SOD1*-type ALS MNs [32]. Therefore, the disease mechanisms and candidate therapeutic targets of *SOD1* and sporadic ALS must still be elucidated.

In the present study, we applied a CHSF-based differentiation method (a neural differentiation protocol with CHIR99021, SB431542, and FGF-basic) for efficient generation of high-purity *SOD1* ALS MNs. The novel paradigmatic nerve fibers and functional cytopathology of ALS were observed in *SOD1* ALS iPSCs but not in corrected healthy iPSC-derived MNs within 28–38 days of differentiation. Gastrodin, a natural small compound screened from this model, was able to reduce nerve fiber and neurofunctional cytopathies, while also providing therapeutic effects on sporadic ALS MNs and transgenic mice. IGF-1 and GSK3β activation were the major therapeutic mechanisms of gastrodin treatment and also significantly reduced ALS phenotypes. Interestingly, the role of GSK3β in ALS reported here is opposed to that previously reported in earlier transgenic model-based studies.

## 2. Materials and Methods

### 2.1. iPSC Line Establishment and Culture

The *SOD1*^D90A^ and *SOD1*^D90D^ iPSC lines were purchased from WiCell (Madison, WI, USA). *SOD1*^G85R^ and sporadic ALS iPSC lines were generated from patients’ PBMCs with the approval of the Ethic Institutional Review Board in Hualien Tzu Chi Hospital (IRB105-131-A). Informed consent was obtained from patients. Patients’ PBMCs were cultured in StemPro 34 SFM (ThermoFisher Scientific, Waltham, MA, USA) supplemented with SCF, FLT-3, IL-3, and IL-6 (all Peprotech, Cranbury, NJ, USA). Reprogramming was processed with a CytoTune iPS 2.0 Sendai Reprogramming Kit [34] (ThermoFisher Scientific) according to the kit manual. Emerging colonies were transferred to 1% Geltrex (ThermoFisher Scientific)-coated culture dishes and expanded in TeSR-E8 medium (StemCell Technologies, Vancouver, BC, Canada) or Pluto human iPS/ES cell culture medium (DuoGenic Stem Cells, Taichung, Taiwan). The iPSCs were passaged for 3–5 days with Accutase (Merck-Millipore, Billerica, MA, USA) and then reseeded at 1:5 to 1:10 ratios. Culture media were refreshed daily.

### 2.2. SOD1^G85R^ iPSC Site-Specific Correction by CRISPR/Cas9-Mediated Homologous Recombination

*SOD1*G85R mutation correction was processed by Applied StemCell (Milpitas, CA, USA). Briefly, gRNAs were designed near exon 4 of *SOD1* and the site-specific cutting efficiency was identified with a surveyor nuclease assay. A 200-nt single-stranded DNA oligonucleotide homologous arm around the *SOD1* G256C mutated site was delivered together with Cas9/sgRNA plasmid by nucleofection. Emerging colonies were individually collected and the *SOD1* G256 site was analyzed by DNA sequencing. Two clones, A7 and A9, were identified with corrected G256G sites. The *SOD1*^G85G^ iPSC line used in this study was the A7 clone.

### 2.3. Robust MN Differentiation from iPSCs

While the iPSCs reached 80% confluence in a culture dish, the cells were treated with 1 mg/mL of Dispase II (Sigma-Aldrich, Saint Louis, MO, USA) for 5–15 min until the periphery of colonies started to become rounded up; they were then lifted by scraping. The cell clumps were dissociated into 200–300 μm clusters and transferred into ultra-low attachment dishes in TeSR-E6 medium (StemCell Technologies) for 1 day to form embryoid bodies (EBs). Additionally, 5 μM of Y27632 (Cayman Chemical, Ann Arbor, MI, USA) was added during the first 4 h to avoid iPSC blebbing. The EBs were subjected to the CHSF neural epithelial induction method. The neural induction medium comprised DMEM-F12 basal medium supplied with 1% N2 supplement, 1 mM of NEAAs, and 2 mM of glutamate (all ThermoFisher Scientific). CHSF factors, including 3-μM CHIR99021 (Cayman Chemical), 10-μM SB431542 (Sigma-Aldrich), and 10-ng/mL recombinant human FGF-2 (rh-FGF2; R&D Systems, Minneapolis, MN, USA) were added on days 1–4. On day 4, the neural induction medium was changed to neurobasal medium (ThermoFisher Scientific) with 1% N2 supplement, 1-mM NEAAs, 2-mM glutamate, and MN patterning factors, including 3 μM of CHIR99021, 2 μM of SB431542, 200 nM of LDN193189 (Cayman Chemical), 0.5 μM of SAG (Cayman Chemical), and 0.5 μM of retinoic acid (Sigma-Aldrich), until day 14 of differentiation. Both 0.5 μM SAG and 0.5 μM retinoic acid were continually added until day 25 of differentiation. According to EB condition, 2% B27 supplement (ThermoFisher Scientific) was potentially added to the culture medium and the EBs were moved to cell culture flasks or dishes on day 15. Before experimental assays, the EBs were dissociated into small clumps using Accutase and seeded on 1% Geltrex-coated cell culture dishes supplemented with 0.2 μM of compound E (Cayman Chemical) but without BDNF, GDNF, or dbcAMP. The addition of neurotrophic factors can delay or eliminate the ALS nerve fiber cytopathies in iPSC-derived MNs. In the first 4 h of cell adhesion, 1% RevitaCell supplement (ThermoFisher Scientific) was added to the cells.

### 2.4. Immunocytochemistry (ICC) Staining and Nerve Fiber Density/Beads Calculations

First, iPSCs and differentiated cells were seeded in 24-well plates and fixed with 4% paraformaldehyde (Sigma-Aldrich) at room temperature; then, they were washed twice with phosphate-buffered saline (PBS). Cells were permeabilized with 0.1% Triton-100 for 15 min at 4 °C and then blocked with 5% horse serum. Primary antibodies were incubated with cells at 4 °C for 16 h, washed twice, and then conjugated with secondary antibodies. The primary antibodies used in this study included those against Oct-4 (1:200), Brachyury (1:50), N-cadherin (1:500), and Sox-1 (1:200) (all Santa Cruz Biotechnology); Nanog (1:30) and Sox-17 (1:20) (both R&D systems); Pax-6 (1:100), Nestin (1:500), and βIII-tubulin (1:500) (all Covance, Princeton, NJ, USA); NF (1:500), phosphorylated neurofilament H & M (1:500), and choline acetyltransferase (1:100) (all Sigma-Aldrich); and Sox-2 (1:200; GeneTex, Irvine, CA, USA), SSEA-4 (1:100; BD Pharmingen, Franklin Lakes, NJ, USA), Islet-1 (1:500; Neuromics, Edina, MN, USA), Oligo-2 (1:500; Novus Biologicals, Centennial, CO, USA), and HB9/MNX1 (1:50; Developmental Studies Hybridoma Bank, DSHB, Iowa City, IA, USA). Cell nuclei were stained with diamidino-2-phenylindole (DAPI). Fluorescence images were captured using an upright microscope (Imager Z1, Zeiss, Oberkochen, Germany). Neurite density and nerve fiber beads were calculated with Olympus CellSens Dimension Desktop 3.1 (Olympus Corporation, Tokyo, Japan) using the images from three independent experiments.

### 2.5. Calcium Imaging

Cells were seeded on Geltrex-coated 10-mm cover slides and cultured in physiological buffer with 1 μM of Fluo-4 at 37 °C for 40 min. Subsequently, they were incubated in physiological buffer (including 30 mM of NaCl, 5 mM of KCl, 2 mM of CaCl_2_, 2 mM of MgCl_2_, 10 of mM glucose, and 10 mM of HEPES) for 20 min. The cells were then moved to a calcium imaging chamber, washed with physiological buffer for 30 s, and treated with 60 mM of KCl for 60 s. After a 5 min wash in physiological buffer, the cells were treated with 1 mM of l-glutamate for 60 s. Images were taken with an ECLIPSE Ti2-E microscope (Nikon, Tokyo, Japan) and analyzed with NIS-Elements AR (Nikon).

### 2.6. ALS Animal Model and Small Compound Treatment

Transgenic mice overexpressing the human *SOD1*^G93A^ mutation (*SOD1*-G93A-1Gur/J) were purchased from Jackson Laboratory (Bar Harbor, ME, USA) and used in this study. All animal-related procedures were performed in accordance with the guidelines of the US National Institutes of Health and Hualien Tzu Chi Hospital (106-46), Taiwan. Mice were separated into three groups at 60 days of age: (1) vehicle control, (2) 50 mg/kg body weight of gastrodin, and (3) 200 mg/kg body weight of gastrodin. They were treated with vehicle or gastrodin by intraperitoneal injection five times per 30 days within the individual animal’s lifespan.

### 2.7. Animal Motor Behavior Test

Motor function of ALS mice was measured using the BBB scale, grip strength test, and rotarod test. BBB scale measurements began at 90 days of age and continued within the individual animal’s lifespan. Briefly, mice were free to walk around their cage and were observed for ~3–5 min. The hindlimb score was based on a 21 to 0-point scoring range from normal to no movement as measured by tail elevation, paw position, sweeping without weight bearing, movement of the hindlimb joints, forelimb–hindlimb step, consistent weight support, and frequent plantar stepping [35]. In the grip strength test, mice were placed on a grid and the base of their tail was pulled in the opposite direction to record their grip power. In the rotarod test, before 90 days of age, mice were placed on a rotating rod that turned at 5 rpm for 300 s for training. After 90 days of age, mouse data were collected by rotating the rod at 5 rpm on average with an acceleration of 0–30 rpm.

### 2.8. Western Blotting

Cells were lysed in a 200-μL lysis buffer composed of M-PER, 1% phosphatase protease inhibitor, and 50 mM of EDTA; subsequently, they were centrifuged at 14,000 rpm and 4 °C for 15 min. Protein concentrations were determined using the Bradford assay (Bio-Rad Laboratories, Hercules, CA, USA). Purified proteins (15 mg) were subjected to SDS-PAGE, transferred to PVDF membranes, blocked with 5% skim milk for 1 h, and then probed with primary antibodies against GAPDH (1:10000), p44/42 MAPK (1:1000), phospho-p44/42 MAPK (Thr202/Tyr204) (1:1000), MEK1/2 (1:1000), phospho-MEK1/2 (Ser217/221) (1:1000), Akt (pan) (1:1000), phospho-Akt (Ser473) (1:2000) (all Cell Signaling Technology, Danvers, MA, USA), GSK3β (1:3000; ProteinTech, Rosemont, IL, USA), and phospho-GSK3 (Tyr279/Tyr216) (1:500; Sigma-Aldrich) at 4 °C overnight. Membranes were then washed with PBS containing 0.1% Tween-20 before they were incubated with HRP-conjugated secondary antibody at room temperature for 1 h. All proteins were detected using Immobilon Crescendo Western HRP substrate (Millipore) and recorded with FUSION Solo S (Vilber, Collégien, France). The band intensity of Western blotting was quantified with image J (National Institutes of Health) then applied to statistical analysis.

### 2.9. RNA-Microarray and Analysis

An amount of 0.2 μg of total RNA was amplified by a Low Input Quick-Amp Labeling kit (Agilent Technologies, Santa Clara, CA, USA) and labeled with Cy3 (CyDye, Agilent Technologies) during the in vitro transcription process. Then 0.6 μg of Cy3-labled cRNA was fragmented by incubation with fragmentation buffer at 60 °C for 30 min. Correspondingly fragmented labeled cRNA was then pooled and hybridized to Agilent SurePrint Microarray (Agilent Technologies) at 65 °C for 17 h. After washing and drying by nitrogen gun blowing, microarrays were scanned with an Agilent microarray scanner (Agilent Technologies). Scanned images were analyzed by Feature extraction10.7.3.1 software (Agilent Technologies), an image analysis and normalization software that is used to quantify signal and background intensity for each feature. Raw signal data were normalized by quantile normalization for differential expressed genes discovering. For functional assay, we provided an enrichment test for differential expressed genes (For most model organisms). Welgen Biotech used clusterProfiler for enrichment test for gene ontology (GO) and pathway (KEGG).

### 2.10. Statistical Analysis

Data were collected and analyzed from no less than three independent experimental results and are shown as means ± SD. Student’s t-test or one-way ANOVA with Tukey’s post hoc test were used to test for differences between groups. *p* values < 0.05 were considered statistically significant.

## 3. Results

### 3.1. Generation of SOD1^G85R^ ALS and SOD1^G85G^ Isogenic iPSCs, and Robust MN Differentiation via the CHSF Method

**Figure 1:**
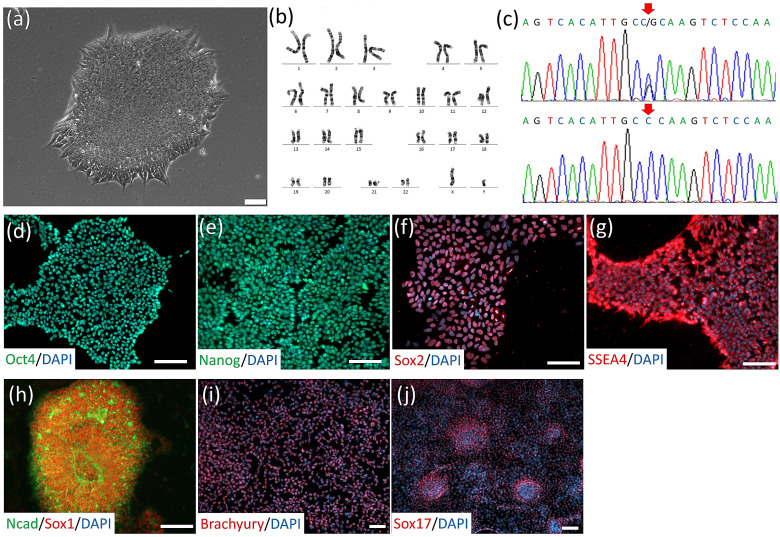
iPSC generation and SOD1 mutant site correction. (**a**) Morphology of *SOD1*^G85R^ ALS patient’s PBMC-derived iPSC clone. (**b**) Karyotype of *SOD1*^G85R^ iPSCs. (**c**) DNA sequencing of *SOD1*^G85R^ iPSCs (upper panel) and corrected *SOD1*^G85G^ iPSCs (lower panel). Mutant and corrected site are emphasized with arrows. (**d**–**g**) *SOD1*^G85R^ iPSC-expressed pluripotent stem cell-specific markers including Oct-4 (**d**), Nanog (**e**), Sox-2 (**f**), and SSEA4 (**g**). (**h**–**j**) In vitro differentiation of *SOD1*^G85R^ iPSCs into cell types with three germ layer cell markers including the ectoderm markers N-cadherin and Sox-1 (**h**), mesoderm marker Brachyury (**i**), and endoderm marker Sox-17 (**j**). Scale bar, 100 μm.

We obtained *SOD1*^D90A^- and *SOD1*^D90D^-corrected isogenic iPSC lines from WiCell, which were previously reported by Chen et al. [27]. Additionally, we established *SOD1*^G85R^ iPSC lines from the peripheral blood mononuclear cells (PBMCs) of an early-onset ALS patient. G85R was one of the earliest discovered *SOD1* mutant sites in familial ALS [36,37]. After reprogramming and clonal selection, individual iPSC clones became stable lines. One clone (*SOD1*^G85R^) was subjected to iPSC characterization. *SOD1*^G85R^ iPSC exhibited classical pluripotent stem cell morphology (Figure 1a) and expressed pluripotent-specific markers including Oct4, Nanog, Sox2, and SSEA-4 (Figure 1d–g). The *SOD1*^G85R^ iPSCs obtained normal karyotypes (Figure 1b) and heterogeneous G to C mutations on the *SOD1* 256-nucleic acid site in exon 4 (Figure 1c, upper panel; the G256C mutation was identified with reverse primer sequencing). After in vitro differentiation, *SOD1*^G85R^ iPSCs could differentiate into cells expressing three germ-layer progenitor cell markers, i.e., ectoderm with Sox1 and N-Cadherin expression (Figure 1h), mesoderm with Brachyury expression (Figure 1i), and endoderm with Sox17 expression (Figure 1j).

For a reliable ALS cytopathy isogenic control, we applied the CRISPR/Cas9 gene editing tool to correct the *SOD1* G256C mutation. The normal *SOD1* sequence near the *SOD1* G256C site was flanked with homologous arms and sgRNAs were delivered into *SOD1*^G85R^ iPSCs. After clonal expansion, the *SOD1* G256C site was analyzed by sequencing and one iPSC clone was identified (*SOD1*^G85G^-A7) with a mutant site correction (Figure 1c, lower panel; homogeneous G was identified with reverse primer sequencing).

**Figure 2:**
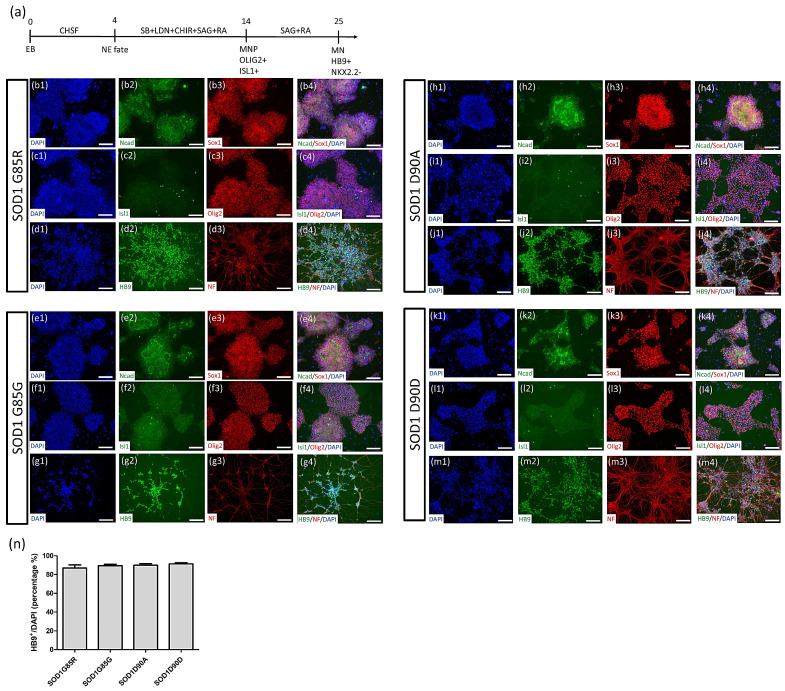
Robust generation of MNs from iPSCs. (**a**) Procedure for CHSF-based MN differentiation. (**b**–**m**) Identification of *SOD1*^G85R^ (**b**–**d**), *SOD1*^G85G^ (**e**–**g**), *SOD1*^D90A^ (**h**–**j**), and *SOD1*^D90D^ (**k**–**m**) iPSC line-derived MN progenitors and MNs. Every line was identified with neural stem cell markers (N-cadherin and Sox-1) and MN progenitor markers (Oligo-2 and Islet-1) at day 15 of differentiation, and with an MN marker (HB9) and a neuronal marker (NF) at days 23–25 of differentiation. (**n**) Calculation of the HB9 expression ratio of DAPI^+^ cells. Scale bar, 100 μm.

Our neural stem cell (NSC) differentiation method was modified from the previously reported BiSF method [38,39]. Specifically, BIO in the BiSF method was replaced with CHIR99021 (a GSK3β inhibitor). After NSC induction, MN patterning factors were added to culturing cells (Figure 2a). At day 15, differentiated cells of four iPSC lines (*SOD1*^G85R^: Figure 2b,c; *SOD1*^G85G^: Figure 2e,f; *SOD1*^D90A^: Figure 2h,i; and *SOD1*^D90D^: Figure 2k,l) expressed the neural stem cell-specific markers Sox1 and N-cadherin as well as the MN progenitor markers Oligo2 or Islet1 in a large number of rosette-like structures. According to the previous publication, the MN progenitor markers Oligo2 or Islet1 might represent the MN fate cells at different MN differentiation stages [40]. Thus, the total cells with the expression of Oligo2 or Islet1 (Figure 2c,f,I,l) presented a high yield of potential MN fate cells at day 15. At days 23–25, differentiated cells expressed neurofilament protein (NF) in nerve fiber-like structures and the MN-specific marker HB9 (also known as MNX1) (*SOD1*^G85R^: Figure 2d; *SOD1*^G85G^: Figure 2g; *SOD1*^D90A^: Figure 2j; and *SOD1*^D90D^: Figure 2m). The expression ratio of HB9 at day 25 was approximately 90–94% of NF^+^ cells (SOD1^G85R^: 90.95 ± 3.61%; SOD1^G85G^: 94.20 ± 1.23%; SOD1^D90A^: 94.33 ± 1.53%; SOD1^D90D^: 92.93 ± 2.35%) and 86%–91% of total cells (SOD1^G85R^: 86.85 ± 4.88%; SOD1^G85G^: 89.47 ± 2.61%; SOD1^D90A^: 89.90 ± 2.69%; SOD1^D90D^: 91.33 ± 2.04%) (Figure 2n) in each line. These data suggest that the *SOD1* mutant and corrected iPSC lines did not have a significant effect on MN differentiation ability.

### 3.2. NF Aggregates and Nerve Fiber Degeneration in SOD1 ALS MNs

**Figure 3:**
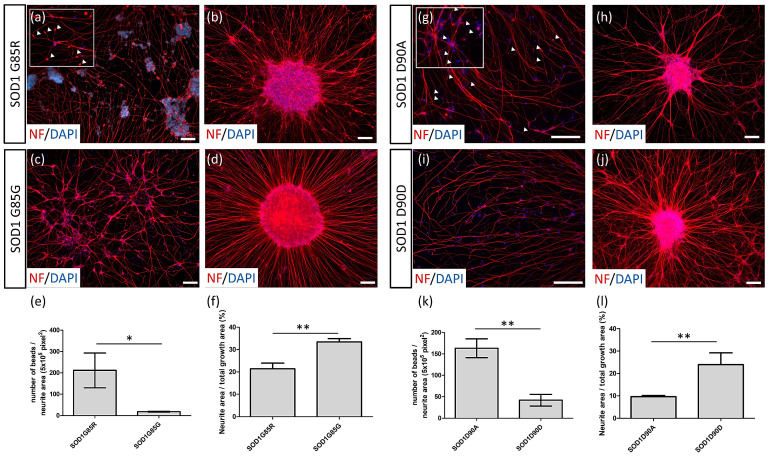
Nerve fiber beads and neurite degeneration in SOD1 ALS MNs. (**a**–**d**) Nerve fiber beads (**a**) and neurite density (**b**) of *SOD1*^G85R^ and *SOD1*^G85G^ (nerve fiber beads: c; neurite density: d) MNs identified with NF immunocytochemistry staining. (**e**) Number of nerve fiber beads of *SOD1*^G85R^ and *SOD1*^G85G^ MNs at day 35 of differentiation. (**f**) Neurite density of *SOD1*^G85R^ and *SOD1*^G85G^ MNs at day 28 of differentiation. (**g**–**j**) Nerve fiber beads (**g**) and neurite density (**h**) of *SOD1*^D90A^ and *SOD1*^D90D^ (nerve fiber beads: (**i**); neurite density: (**j**)) MNs. (**k**) Number of nerve fiber beads of *SOD1*^D90A^ and *SOD1*^D90D^ MNs at day 35 of differentiation. (**l**) Neurite density of *SOD1*^D90A^ and *SOD1*^D90D^ MNs at day 28 of differentiation. The nerve fiber beads are indicated by arrow heads. * *p* < 0.05 and ** *p* < 0.01. Scale bar, 100 μm.

NF inclusions in the MNs of ALS patient tissue have previously been identified; NF inclusions in *SOD1*^D90A^ and *SOD1*^A4V^ MNs were first discovered by Chen et al. [27]. In the present study, we found NF inclusions in *SOD1*^G85R^ MNs in somas and bead-like aggregates on neurites (211.4 ± 47.11 beads/5 × 10^5^ pixels^2^ NF) (Figure 3a) but not in the *SOD1*^G85G^ control (18.23 ± 1.13 beads/5 × 10^5^ pixels^2^ NF) (Figure 3c; calculated as Figure 3e), similar to the findings for *SOD1*^D90A^ MNs (*SOD1*^D90A^: 163.3 ± 12.70 beads/5 × 10^5^ pixels^2^ NF; *SOD1*^D90D^: 41.92 ± 7.76 beads/5 × 10^5^ pixels^2^ NF) (Figure 3g,i,k). For the non-MN control, the Shh agonist SAG was replaced with the antagonist cyclopamine to generate neuron types in the dorsal spinal cord; no obvious NF inclusions were observed in *SOD1*^G85R^ and *SOD1*^D90A^ non-MNs (data not shown).

The NF^+^ neurite density of *SOD1*^G85R^ MNs was reduced 36% (21.4% ± 1.45% of total growth area) (Figure 3b) relative to that of the *SOD1*^G85G^ control (33.43% ± 0.84% of total growth area) (Figure 3d). These data were calculated as Figure 3f. The neurite density of *SOD1*^D90A^ MN was reduced 60% (9.63% ± 0.29% of total growth area) (Figure 3h) relative to that of the *SOD1*^D90D^ control (23.90% ± 3.05% of total growth area) (Figure 3j). The data were calculated as Figure 3l. These data illustrate that we faithfully recapitulated NF aggregates in *SOD1*^D90A^ MNs, but also discovered similar NF inclusions in *SOD1*^G85R^ MNs for the first time. Moreover, we observed a decrease in NF^+^ neurites in both *SOD1* mutant MNs, which could be a novel marker for quantification of ALS-related MN degeneration cytopathies in drug screening.

### 3.3. Gastrodin Reduces NF Aggregates and Promotes Nerve Fiber Extension in SOD1 ALS MNs

**Figure 4:**
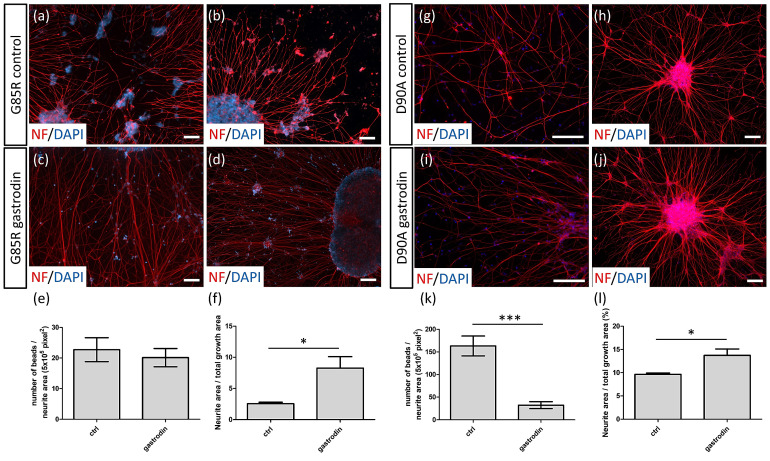
Gastrodin reduces nerve fiber abnormalities of SOD1 ALS MNs. (**a**–**d**) Nerve fiber beads (**a**) and neurite density (**b**) of untreated and gastrodin-treated *SOD1*^G85R^ (nerve fiber beads: c; neurite density: d) MNs identified with NF immunocytochemistry staining. (**e**) Number of nerve fiber beads of untreated (ctrl) and gastrodin-treated *SOD1*^G85R^ MNs at day 35 of differentiation. (**f**) Neurite density of untreated (ctrl) and gastrodin-treated *SOD1*^G85R^ MNs at day 28 of differentiation. (**g**–**j**) Nerve fiber beads (**g**) and neurite density (**h**) of untreated and gastrodin-treated *SOD1*^D90A^ (nerve fiber beads: (**i**); neurite density: (**j**)) MNs. (**k**) Number of nerve fiber beads of untreated (ctrl) and gastrodin-treated *SOD1*^D90A^ MNs at day 35 of differentiation. (**l**) Neurite density of untreated (ctrl) and gastrodin-treated *SOD1*^D90A^ MNs at day 28 of differentiation. * *p* < 0.05, and *** *p* < 0.001. Scale bar, 100 μm.

After the establishment of an in vitro ALS MN model with quantifiable cytopathies, we applied several natural compounds with neuroprotective effects in drug tests. Results showed that gastrodin, a major ingredient of the natural herb *Gastrodia eleta* previously reported to benefit spinal cord injury [41], had therapeutic effects on ALS MNs. After 72 h of gastrodin treatment, the nerve fiber beads of *SOD1*^G85R^ MNs (Figure 4c) were decreased but not significantly so in comparison to the control (Figure 4a; calculated as Figure 4e). However, the *SOD1*^G85R^ MN neurite area was increased by 323% (from 2.56% ± 0.16% to 8.27% ± 1.06% of total growth area; Figure 4f) after gastrodin treatment (gastrodin: Figure 4d; control: Figure 4b).

Gastrodin also showed therapeutic effects on *SOD1*^D90A^ nerve fiber cytopathies. After treatment, the number of NF aggregates decreased from 163.3 ± 12.70 (control: Figure 4g) to 32.38 ± 4.39 beads/5 × 10^5^ pixels^2^ NF (gastrodin: Figure 4i, calculated as Figure 4k). The number of nerve fiber aggregates was reversed to the basal level, similar with that of *SOD1*^D90D^ healthy MNs (41.92 ± 7.76 beads/5 × 10^5^ pixels^2^ NF) (Figure 3k) after gastrodin treatment, illustrating the therapeutic effect of gastrodin on the NF inclusions of *SOD1*^D90A^ MNs. Gastrodin treatment also increased *SOD1*^D90A^ MN nerve fibers by 42.58%, from 9.63% ± 0.29% (control: Figure 4h) to 13.73% ± 1.37% of total growth area (gastrodin: Figure 4j; calculated as Figure 4l). These results support the therapeutic potential of gastrodin against both *SOD1* ALS nerve fiber phenotypes.

### 3.4. Gastrodin Reverses the Glutamate Induced Hyper-Calcium Flux in SOD1 ALS MNs

**Figure 5:**
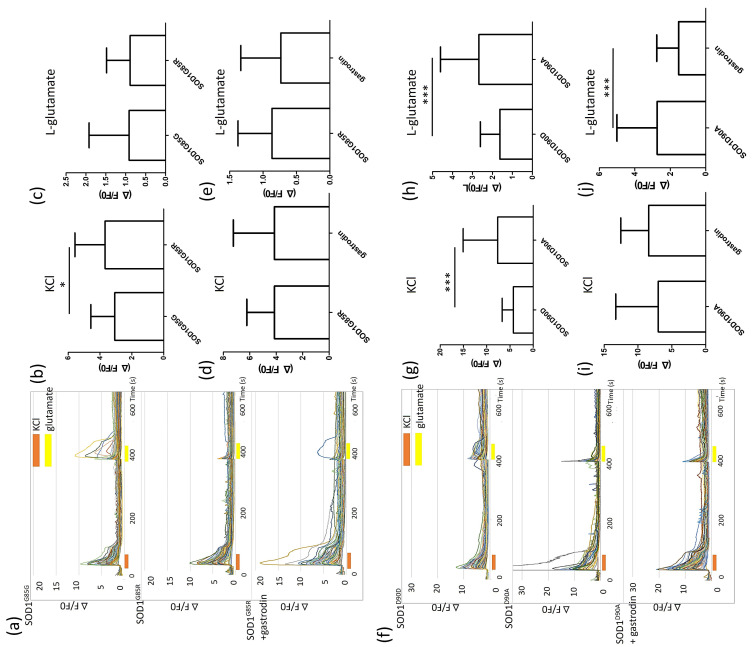
Gastrodin reverses SOD1^D90A^ MN hyper-calcium flux after glutamate stimulation. (**a**) *SOD1*^G85G^, *SOD1*^G85R^, and gastrodin-treated *SOD1*^G85R^ MNs were subjected to Fluo-4 calcium imaging at day 38 of differentiation. The calcium flux stimulated by potassium chloride (presented as orange bars) and glutamate (yellow bars) was revealed with ΔF/F0. (**b**,**c**) Average calcium flux stimulated by potassium chloride (**b**) and glutamate (**c**) of *SOD1*^G85G^ and *SOD1*^G85R^ MNs. (**d**,**e**) Average calcium flux stimulated by potassium chloride (**d**) and glutamate (**e**) of untreated and gastrodin-treated *SOD1*^G85R^ MNs. (**f**) *SOD1*^D90D^, *SOD1*^D90A^, and gastrodin-treated *SOD1*^D90A^ MNs were subjected to calcium imaging. (**g**,**h**) Average calcium flux stimulated by potassium chloride (**g**) and glutamate (**h**) of *SOD1*^D90D^ and *SOD1*^D90A^ MNs. (**i**,**j**) Average calcium flux stimulated by potassium chloride (**i**) and glutamate (**j**) of untreated and gastrodin-treated *SOD1*^D90A^ MNs. * *p* < 0.05, *** *p* < 0.001.

Identifying neuronal electrophysiological function markers is important for linking in vitro models with real-world ALS MN functional degeneration in patients. According to calcium image tests, *SOD1*^G85R^ MNs showed significantly higher calcium flux (ΔF/F0: 3.68 ± 0.21) than did *SOD1*^G85G^ MNs (ΔF/F0: 3.07 ± 0.14) with potassium chloride treatment but not with glutamate stimulation (Figure 5a–c). Additionally, *SOD1*^D90A^ MNs showed higher calcium flux (ΔF/F0: 7.65 ± 1.08) than did *SOD1*^D90D^ MNs (ΔF/F0: 4.26 ± 0.23) with potassium chloride treatment. After glutamate triggering, *SOD1*^D90A^ MNs showed higher calcium influx (ΔF/F0: 2.68 ± 0.28) than did *SOD1*^D90D^ MNs (ΔF/F0: 1.63 ± 0.09) (Figure 5f–h). Both *SOD1* ALS MNs expressed hyper-calcium flux to potassium ions or glutamate stimulation, which is similar to hyperexcitability cytopathy that has been reported for ALS patients [42], but this is first report from an iPSC-based model.

After gastrodin treatment, the calcium influx of *SOD1*^D90A^ MNs decreased from 2.74 ± 0.32 to 1.53 ± 0.13 after glutamate stimulation and reversed the calcium flux back to a level similar to that of *SOD1*^D90D^ (ΔF/F0: 1.63 ± 0.09) (Figure 5j). In contrast, gastrodin treatment did not alter the potassium and glutamate-induced calcium flux of *SOD1*^G85R^ (Figure 5d,e), or the potassium-induced calcium flux of *SOD1*^D90A^ MNs (Figure 5i).

### 3.5. Gastrodin Rescues Nerve Fiber Cytopathies in Sporadic ALS MNs As Well As Restoring Motor Performance and Prolonging Survival in ALS Mice

**Figure 6:**
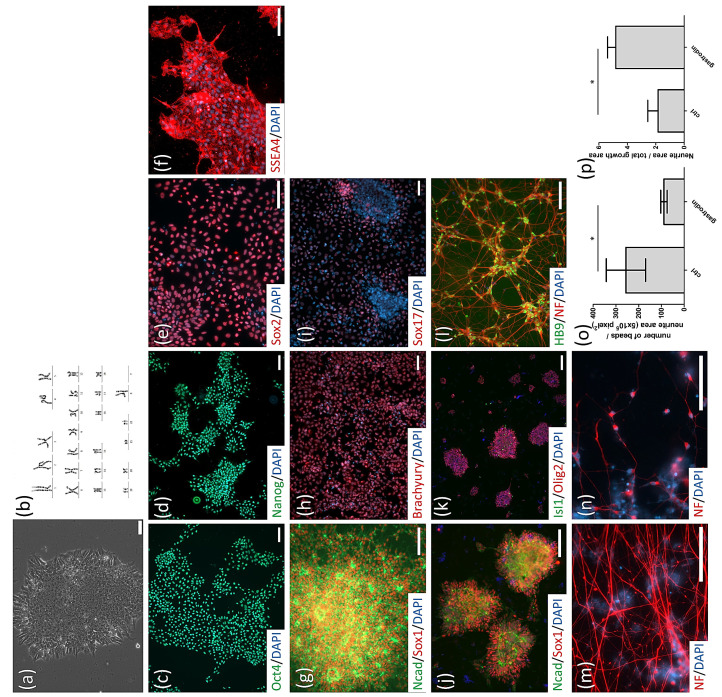
Gastrodin reverses nerve fiber degeneration in sporadic ALS MNs. (**a**) Morphology of sporadic ALS (sALS) iPSC clones. (**b**) Karyotype of sALS iPSCs. (**c**–**f**) sALS iPSC-expressed pluripotent stem cell markers including Oct-4 (**c**), Nanog (**d**), Sox-2 (**e**), and SSEA4 (**f**). (**g**–**i**) In vitro differentiation of sALS iPSCs into three germ layer cell types with markers including the ectoderm markers N-cadherin and Sox-1 (**g**), mesoderm marker Brachyury (**h**), and endoderm marker Sox-17 (**i**). (**j**–**l**) sALS iPSC line-derived MNs were identified with neural stem cell markers (N-cadherin and Sox-1) and MN progenitor markers (Oligo-2 and Islet-1) at day 15 of differentiation, and with an MN marker (HB9) and a neuronal marker (NF) at day 25 of differentiation. (**m**,**n**) Nerve fiber beads (**m**) and cell body inclusions (**n**) of NF protein on sALS MNs. (**o**,**p**) Number of nerve fiber beads (**o**) and neurite intensity (**p**) of untreated (ctrl) and gastrodin-treated sALS MNs at day 62 of differentiation. * *p* < 0.05. Scale bar, 100 μm.

To evaluate the therapeutic potential of gastrodin in non-*SOD1*-mutated ALS MNs, PBMCs were collected from a sporadic ALS patient (sALS), i.e., without known ALS mutations on *SOD1*, *TARDBP*, *c9orf72*, *VAPB*, and *FUS*, and reprogrammed to iPSCs. sALS iPSCs presented classical pluripotent stem cell morphology (Figure 6a), normal karyotypes (Figure 6b), and pluripotent-specific markers (Figure 6c–f). According to in vitro differentiation tests, sALS iPSCs could differentiate into cell lineages with the expression of ectoderm (Figure 6g), mesoderm (Figure 6h), and endoderm (Figure 6i) progenitor markers.

sALS iPSCs differentiated into MNs with high efficiency. NSC markers (sox1 and N-cadherin) and MN progenitor markers (Islet1 and Oligo2) were expressed at day 15 of differentiation, whereas MN markers (HB9 and NF) were expressed at day 28 (Figure 6j–l). Nerve fiber cytopathies of sALS MNs arose around day 60 of differentiation: nerve fibers showed abnormal NF aggregates on neurites (Figure 6m), inclusions on somas, and nerve fiber fragmentations (Figure 6n). After 72 h of gastrodin treatment, the number of nerve fiber beads decreased from 256.2 ± 49.81 beads/5 × 10^5^ pixels^2^ NF to 89.17 ± 9.79 beads/5 × 10^5^ pixels^2^ NF (Figure 6o), whereas the amount of nerve fibers increased from 1.84% ± 0.51% to 4.82% ± 0.41% of the total growth area (Figure 6p), i.e., approximately 2.6-fold that of untreated sALS MNs. These results demonstrate that gastrodin can rescue neurite cytopathies not only in *SOD1* mutants but also in sALS MNs.

**Figure 7:**
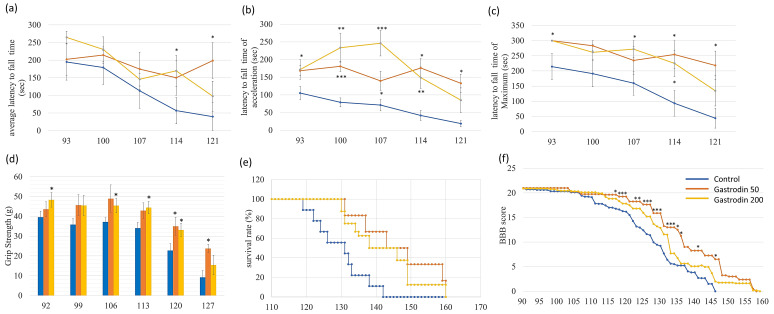
Gastrodin improves the motor function and prolongs the life span of SOD1^G93A^ ALS mice. ALS mice were treated with gastrodin from 60 days of age. Data on the rotarod test (**a**–**c**), grip strength (**d**), survival rate (**e**), and BBB score (**f**) were recorded from 90 days-of-age. Rotarod data are presented as the average latency to fall time (**a**), latency to fall time of acceleration (**b**), and latency to fall time of maximum (**c**). * *p* < 0.05, ** *p* < 0.01, and *** *p* < 0.001.

*SOD1*^G93A^ ALS mice were used to evaluate the in vivo therapeutic effects of gastrodin. Gastrodin was delivered from 60 days-of-age at 50 or 200 mg/kg by intraperitoneal injection five times per month. Results of the rotarod and paw force experiments showed that the motor function of ALS mice was significantly increased after gastrodin treatment (Figure 7a–d). The survival period was prolonged by gastrodin: 129.67 ± 7.57 days to 146.5 ± 11.73 (50 mg/kg) or 140.38 ± 10.43 (200 mg/kg) days (both *p* < 0.05; Figure 7e). Gastrodin also improved motor function [Basso–Beattie–Bresnahan (BBB) score] at 120–148 days-of-age (Figure 7f). Thus, the therapeutic effects of gastrodin on ALS were also demonstrated in an animal model.

### 3.6. Gastrodin Activates the IGF-1 Signaling Pathway and GSK3β Activity to Rescue Nerve Fiber Cytopathies in SOD1 ALS MNs

**Figure 8:**
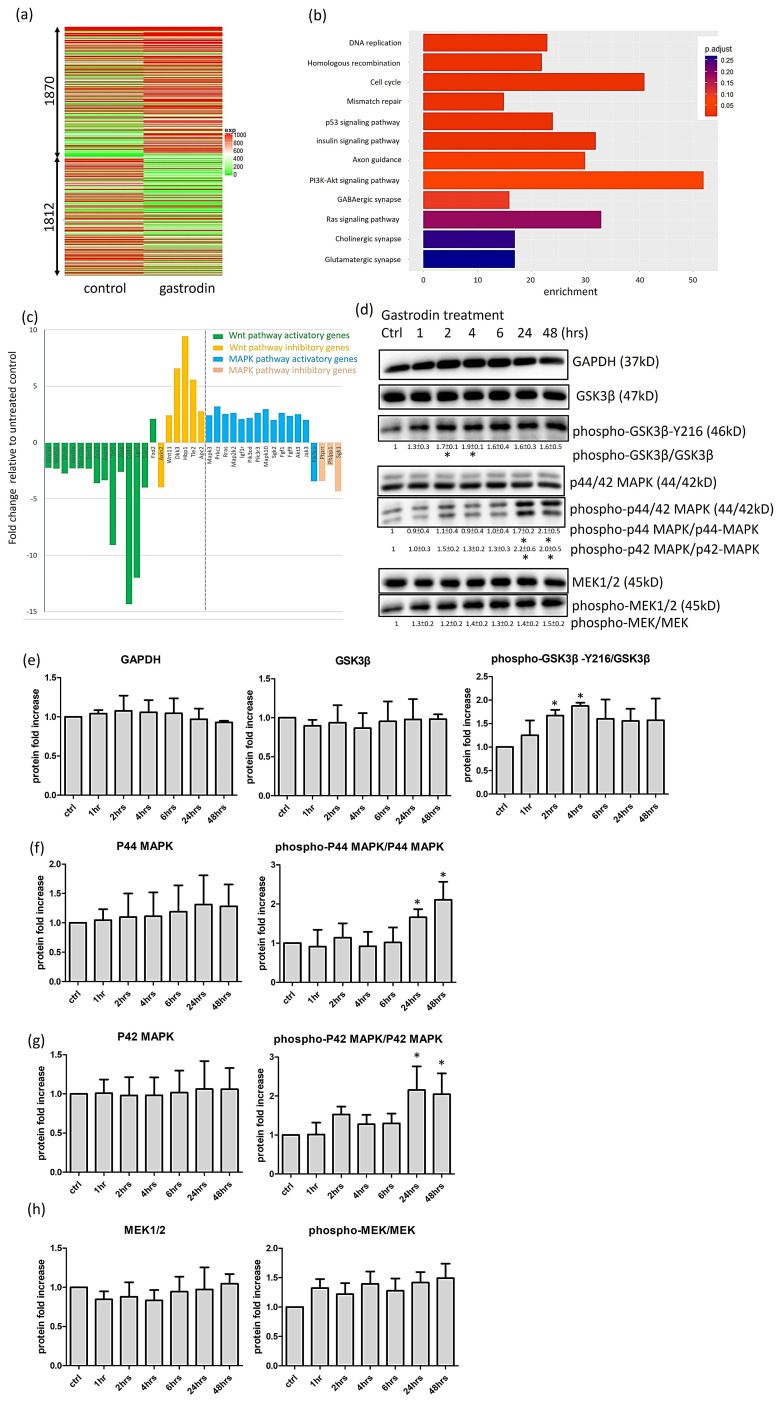
Gastrodin promotes GSK3β and MAPK pathway activation in SOD1^G93A^-NSC34 MN-like cells. (**a**) Dendrogram showing clustering of gastrodin-treated and untreated *SOD1*^G93A^-NSC34 cells based on transcriptional changes detected by RNA-microarray. (**b**) Gene sets enrichment in gastrodin-treated *SOD1*^G93A^-NSC34 cells. (**c**) Graph of expression fold change for Wnt and MAPK signaling-related genes in gastrodin-treated and untreated control *SOD1*^G93A^-NSC34. (**d**–**h**) Western blotting of GAPDH, GSK3β, phospho-GSK3β, MAPK, phospho-MAPK, MEK, and phospho-MEK proteins from gastrodin-treated and untreated (ctrl) *SOD1*^G93A^-NSC34 cells. * *p* < 0.05.

To elucidate the mechanism underlying the therapeutic effect of gastrodin on ALS, we employed an *SOD1*^G93A^-NSC34 ALS cell model with gastrodin treatment. RNA microarray results revealed that 1870 and 1812 genes were up and downregulated after gastrodin treatment (Figure 8a). Enriched genes were clustered into cell cycle, DNA repair, axon guidance, synapse functions, and signaling pathways including p53, insulin, PI3K-Akt and Ras (Figure 8b). Gene expression of most IGF-1 related downstream pathways, including the IGF-1 receptor, MAPK and PI3K-Akt signaling, were upregulated, whereas Wnt signaling was downregulated after gastrodin treatment (Figure 8c). We confirmed that GSK3β was activated after 2–4 h of gastrodin treatment (the phospho-GSK3β/GSK3β were 1.67 ± 0.12 fold at 2 h and 1.87 ± 0.07 fold at 4 h related to ctrl, *p* < 0.05) (Figure 8d), consistent with Wnt pathway downregulation. The MAPK signaling pathway was activated after gastrodin treatment and P42/P44 phosphorylation of MAPK was significantly increased after 24–48 h of treatment (the phospho-p44-MAPK/p44-MAPK were 1.66 ± 0.21 fold at 24 h and 2.11 ± 0.46 fold at 48 h related to ctrl, *p* < 0.05; the phospho-p42-MAPK/p42-MAPK were 2.16 ± 0.60 fold at 24 h and 2.05 ± 0.54 fold at 48 h related to ctrl, *p* < 0.05) (Figure 8d–h). The quantified protein fold changes of different time points were list below every protein group of Figure 8d and presented in Figure 8e–h.

**Figure 9:**
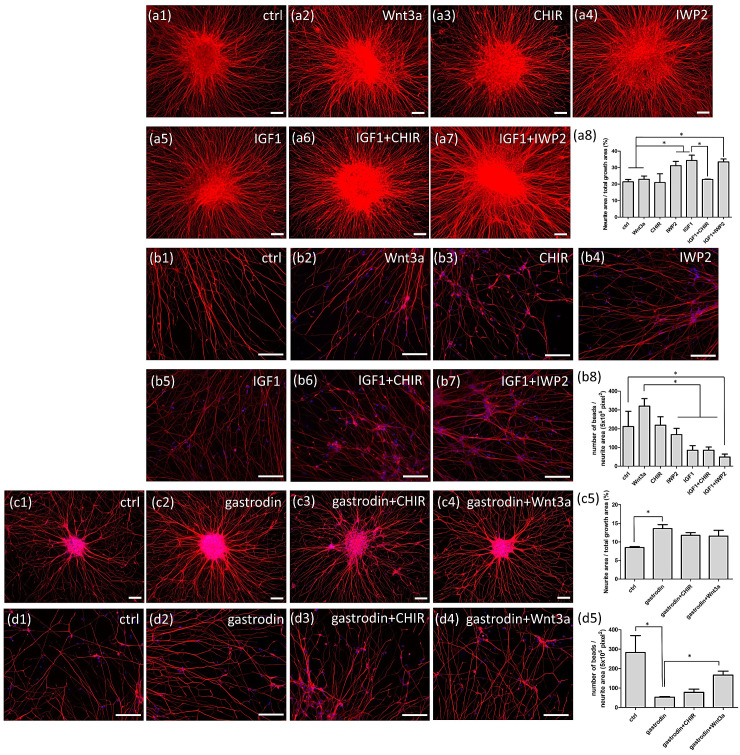
Activation of the GSK3β and IGF-1 pathway reduces axonal cytopathies in SOD1 mutant ALS MNs. (**a**) Neurite densities of day-28 *SOD1*^G85R^ MNs treated with Wnt3a, CHIR99021, IWP-2, and IGF-1, and their combinations were analyzed with NF immunocytochemistry staining and calculated as (**a8**). (**b**) Nerve fiber beads of day-35 *SOD1*^G85R^ MNs treated with the aforementioned compounds and their combinations were analyzed and calculated as (**b8**). (**c**) Neurite density of day-28 *SOD1*^D90A^ MNs treated with gastrodin and its combinations with CHIR99021 or Wnt3a, were analyzed and calculated as (**c5**). (**d**) Nerve fiber beads of day-35 *SOD1*^D90A^ MNs treated with gastrodin and combinations were analyzed and calculated as (**d5**). * *p* < 0.05. Scale bar, 100 μm.

To investigate the effect of GSK3β and IGF-1 coactivation on ALS therapy, *SOD1*^G85R^ iPSC-derived MNs were treated with ligands or inhibitors of candidate signaling pathways, including Wnt3a (Wnt ligand, as an indirectly GSK3β inhibitor), CHIR99021 (GSK3β inhibitor), IWP-2 (Wnt receptor antagonist, as an indirectly GSK3β activator), and IGF-1. Immunofluorescence of neurite intensity showed that compared with control (Figure 9(a1)), IGF-1 (Figure 9(a5)) and IWP-2 (Figure 9(a4)) treatments enhanced ALS MN neurite growth, whereas cotreatment with IGF-1 and IWP-2 did not show additional effects compare with individual treatments (Figure 9(a7)). Wnt3a and CHIR99021 did not alter the amount of neurites in ALS MNs (Figure 9(a2,a3), but they reversed the therapeutic function of IGF-1 (Figure 9(a6)) and showed negative effects of GSK3β inhibition on ALS MN neurite extension. Overall, these data were calculated as Figure 9(a8). For nerve fiber bead cytopathy, IWP-2 (Figure 9(b4)) or IGF-1 (Figure 9(b5)) treatments decreased bead number, but not significantly relative to the control (Figure 9(b1)). Cotreatment with IWP-2 and IGF-1 (Figure 9(b7)) significantly decreased the nerve fiber bead number of *SOD1*^G85R^ ALS MNs, indicating a synergistic effect compared with that of IWP-2 or IGF-1 alone. Potentially, IWP-2 and IGF-1 cotreatment had a superior therapeutic effect on nerve fiber beads to that of gastrodin (Figure 4e). Wnt3a treatment increased nerve bead numbers (Figure 9(b2)), whereas CHIR99021 did not increase bead numbers but estimated with increased beads size (bead size estimated but not measured) (Figure 9(b3,b6)). Overall, these data were calculated as Figure 9(b8).

To evaluate the influence of GSK3β inhibition on the effect of gastrodin, we combined gastrodin with Wnt3a or CHIR99021 to treat MNs. With gastrodin treatment, the amount of nerve fibers significantly increased compared with that of the control (Figure 9(c2,c1)); however, compared with the effects of gastrodin alone, combined treatments with either Wnt3a (Figure 9(c4)) or CHIR99021 (Figure 9(c3)) did not significantly alter nerve fiber amounts. Thus, Wnt3a and CHIR99021 did not alter the therapeutic effect of gastrodin on nerve fiber density (Figure 9(c5)). In contrast, the number of nerve fiber beads significantly decreased after gastrodin treatment (Figure 9(d1,d2)) but re-increased after CHIR99021 (Figure 9(d3)) or Wnt3a (Figure 9(d4,d5)) cotreatment.

These results suggest that coactivation of IGF-1 and GSK3β could alleviate nerve fiber cytopathies and produce even better therapeutic effects than gastrodin. The addition of GSK3β blocker eliminates the effects of gastrodin on rescuing nerve fiber disease markers. Thus, we conclude that IGF-1 and GSK3β activation have positive effects on ALS therapy.

## 4. Discussion

In the present study, we modified the BiSF method [38] by replacing BIO with CHIR99021 for NSC induction and then used MN patterning based on the method of Du et al. [43]. By combining these two processes, we received ~90% of MNs from at least five ALS-related iPSC lines within 23–25 days of differentiation. Measurable nerve fiber beads and neurite degeneration cytopathies emerged within 28–38 days, confirming that this model is stable and scalable as an iPSC-based platform for ALS drug screening. We selected the candidate compound gastrodin according to its ability to improve nerve fiber cytopathies and show supportive effects on neurofunctional recovery; thus, nerve fiber phenotypes seem to be representative early-stage markers and are highly correlated with late-stage neuronal functional loss in ALS. Therefore, using early-stage nerve fiber cytopathies as first-phase screening markers before confirming with late-stage functional markers could be an effective strategy for ALS therapeutic candidate compound screening.

In neurofunctional tests of ALS MNs lasting > 60 days, we found that the hyperexcitation similar properties of *SOD1* ALS MNs arose at around day 38 of differentiation. Interestingly, in neurofunctional tests after day-60 of differentiation, both *SOD1* ALS MNs (*SOD1*^G85R^ and *SOD1*^D90A^, but not SOD1^G85G^ and SOD1^D90D^) triggered 488-nm fluorescence signals, which were followed by cell death immediately after glutamate treatment. Thus, hyperexcitability might worsen and finally cause MN death after normal levels of neurotransmitter stimulation in aging *SOD1* ALS MNs.

Nerve fiber beads not only presented in *SOD1*^G85R^ and *SOD1*^D90A^ ALS MNs but also in one sALS MN, demonstrating that nerve fiber cytopathies were not only restricted to *SOD1*-type ALS. Gastrodin treatment rescued nerve fiber beads and promoted neurite growth in sALS MNs, indicating the therapeutic potential of gastrodin on sALS.

In ALS patients and experimental animals, riluzole can prolong lifespan at the extremely late stage of disease, but it has minor effects on motor function and life quality maintenance [44,45]. Conversely, we demonstrated that the therapeutic function of gastrodin occurs largely in early-stage ALS, not only in an iPSC-based cell model but also in ALS mice. Gastrodin significantly decreased the speed of motor functional degeneration at 90 days of age and delayed the BBB score decline at 120–148 days-of-age; thus, it clearly had effects on early motor function maintenance. Consequently, combining gastrodin and riluzole may be a potential ALS therapeutic strategy worthy of further investigation.

GSK3β is a potential target for ALS therapy; indeed, MN samples from transgenic ALS animals express higher levels of GSK3β activation than normal [46]. Inhibition of GSK3β signaling may also benefit ALS cells or animals [47,48,49,50,51,52]. Additionally, the indirect GSK3β inhibitor of lithium has been applied in clinical trials [53]. However, we found that GSK3β played a converse role in ALS patient iPSC-derived MNs. Specifically, the inhibition of GSK3β by Wnt3a or CHIR99021 aggravated nerve fiber cytopathies, whereas IWP-2 treatment (an indirectly GSK3β activator) rescued MNs from these nerve fiber cytopathies. Interestingly, a recent study demonstrated a similar role for GSK3β in an *SOD1*^E100G^ iPSC-based ALS model: Bhinge et al. (2017) found that XAV-939 (a tankyrase inhibitor, also as an indirectly GSK3β activator) treatment postponed the death of *SOD1*^E100G^ MNs [33]. In another study, the overexpression of Wnt signaling-related ligands and receptors was observed in ALS patient spinal cord samples [54]. Thus, we suggest that the contrary role of GSK3β shown in our iPSC-based results compared with that indicated in previous non-patient-derived ALS cell or animal results demonstrates the major value of using patient cell-derived MNs as a model.

How *SOD1* mutations influence nerve fiber dynamics in ALS MNs is not yet fully clear. A previous report [27] indicated that the D90A SOD1 protein interacts with the UTR region of NF light chain (*NF-L*) mRNA to reduce protein production, which then influences NF assembly, and finally causes ALS nerve fiber cytopathies. The shortage of NF-L protein expression also alters the stability and phosphorylation of NF heavy chain (NF-H) and NF medium chain (NF-M). After supplying NF-L with a Tet-on inducible knock-in, the researchers demonstrated a reduction in nerve fiber cytopathies. This work provided evidence to link *SOD1* mutations with ALS NF cytopathy. Here, we identified misfolded SOD1 aggregates in *SOD1*^G85R^ MNs (data not shown) but they did not colocalize with NF protein aggregates or inclusions, suggesting that mutant SOD1 protein may not directly interact with NF protein. The phosphorylation of NFs, including NF-H, NF-M, and NF-L, plays important roles in NF stability, transport, and assembly, especially at phosphorylation sites on the C-terminus. MAPK and GSK3β are phosphate donors for essential NF phosphorylation [55,56]. For example, the major activation sites of NF-H protein are located on the proximal C-terminus. With the phosphorylation of these activation sites by GSK3β, MAPK, and cdk5, the NF-H protein switches from a closed to open configuration that is capable of transport and neurite assembly. With an inhibitory mutation (S493A) at the GSK3β phosphorylation site, the NF-H protein forms inclusions in the cell body and induces expression of NF aggregates and fragmented neurites [55]; this is consistent with the *SOD1* ALS MN nerve fiber cytopathies in our experiment. Thus, apart from NF-L resupply, we speculate that activation of MAPK and GSK3β may rescue SOD1-induced nerve fiber phenotypes by providing sufficient phosphorylation activities at specific essential sites, which benefits NF protein transport and assembly to eliminate nerve fiber inclusions and promote neurite extension. However, the specific GSK3β-dependent phosphorylation sites and their antibodies on NFs must still be identified and developed.

Although both G85R and D90A *SOD1* mutant MNs had NF inclusions and degeneration cytopathies, the level of these cytopathies differed between the two lines. Compared with D90A MNs, G85R *SOD1* MNs expressed heavier nerve fiber beads, milder neurite degeneration, and less hyperexcitability, suggesting that the *SOD1* mutation site may alter the individual cytopathy levels. Evidently, further research is required to identify the mechanisms underlying the *SOD1* mutant site’s effects on ALS MN cytopathies.

In addition, how gastrodin functions with the IGF-1 pathway and GSK3β activity is not yet clear. Indeed, the role of gastrodin in these pathways has not previously been reported. In living cells or animals, gastrodin would be converted into the secondary metabolite p-hydroxybenzyl alcohol within hours [57]; however, there is no evidence to date that p-hydroxybenzyl alcohol can promote IGF-1 or GSK3β. Further research should therefore be conducted to determine the working mechanisms of gastrodin.

## 5. Conclusions

In conclusion, we established a robust method by which to differentiate iPSCs into MNs (~90%). Consequently, within 38 days of differentiation, we were able to identify ALS-specific cytopathies including neurite degeneration, nerve fiber inclusions, and hyperexcitability in *SOD1* MNs that matched several clinical pathologies of patients. Although the nerve fiber beads of *SOD1*^D90A^ MNs were previously reported by Chen et al. [27], we are the first to report them in *SOD1*^G85R^ MNs. Moreover, both the nerve fiber degeneration and neurotransmitter hyperexcitability of *SOD1*^G85R^ and *SOD1*^D90A^ MNs were reported for the first time here. Gastrodin had therapeutic effects that rescued nerve fiber and neurofunctional cytopathies in ALS MNs while also improving the motor function of ALS animals. IGF-1 and GSK3β, both activated by gastrodin, showed therapeutic effects on nerve fiber cytopathies, demonstrating the potential of their coactivation as an ALS therapy.

## Data Availability

Data supporting the findings of this study are available within the figures and the results section. Further data are available from the corresponding author upon reasonable request.
